# Mr. Ernest Hart

**Published:** 1898-02

**Authors:** 


MR. ERNEST HART, editor of the *British Medical Journal*, died at his residence in London, January 7, 1898, aged 62 years. His early education was obtained at the City of London school, and his medical education at St. George's hospital medical school, and he was admitted to membership in the Royal College of Surgeons in 1856. Soon afterward he turned his attention to literary work and was in succession assistant editor of the *Lancet*, supervising editor of the *Sanitary Record* and of the *London Medical Recorder*, and finally was appointed editor of the *British Medical Journal* in 1866, holding the latter place until his death. The American profession will recall Mr. Hart's visit to this country in 1893, when he attended the meeting of the American Medical Association at Milwaukee, during which time he addressed the American Medical Editors' Association, and also read a paper in the general session of the American Medical Association on water-borne diseases. Coming again to America in the same year he attended the first Pan-American Medical Congress at Washington, in September, 1893.

A few months ago Mr. Hart underwent amputation of a leg for necrosis complicating diabetes, from which he made a temporary rally, but finally succumbed to the onward progress of the malady. Mr. Hart's fame will largely rest upon the fact that during his editorship of the *British Medical Journal* he lifted it from obscurity to fame and made it one of the greatest weekly medical newspapers of the age.

